# Role of vitamin D serum levels in prevention of primary and recurrent melanoma

**DOI:** 10.1038/s41598-021-85294-3

**Published:** 2021-03-12

**Authors:** M. Lombardo, A. Vigezzi, G. Ietto, C. Franchi, V. Iori, F. Masci, A. Scorza, S. Macchi, D. Iovino, C. Parise, G. Carcano

**Affiliations:** 1 Department of Dermatology, ASST Dei Sette Laghi, Varese, Italy; 2 Department of General, Emergency and Transplants Surgery, Università Dell’Insubria, ASST Dei Sette Laghi, Varese, Italy

**Keywords:** Skin cancer, Cancer, Biomarkers

## Abstract

Patients afflicted with melanoma show lower vitamin D serum levels (VDSL) than the healthy population. This hypothesis agrees with its well-known antiproliferative features. An observational study was carried out to collect VDSL in patients suffering from melanoma. Our aim was to identify a potential connection between low VDSL and the risk to incur melanoma. Furthermore, we studied the association between VDSL at the diagnosis of melanoma and other germane prognostic factors. The population held in regard was composed of 154 patients with a diagnosis of melanoma between 2016 and 2019. These patients were retrospectively collected from our follow-up storage. We compared VDSL to clinical and pathological parameters (age, sex, tumour location, Breslow’s depth, Clark’s level, histological subtype, ulceration, et aliqua). Moreover, we recruited a control group with negative melanoma history. Mean and median of VDSL were significantly lower in the melanoma group. Instead, we found a negative association between melanoma and VDSL > 30 ng/L (OR 0.11; *p* < 0.0001). No correlation between VDSL and both Breslow’s depth and Clark’s level was discovered, but the VDSL comparison between *thin* (depth ≤ 1 mm) and *thick* tumours (depth > 1 mm) revealed a statistically significant difference (21.1 ± 8.2 ng/L vs 17.8 ± 8.1; *p* = 0.01). Moreover, VDSL were significantly lower in melanomas with mitotic rate ≥ 1/mm^2^ (22.1 ± 8.3 ng/L; *p* < 0007). Nevertheless, no connection was found between VDSL and both ulceration and positive sentinel nodes (*p* = 0.76; *p* = 0.74). Besides, our study revealed no association between VDSL and histological subtype (*p* = 0.161). Lower VDSL correlate with thick and high mitotic rate tumours. Future prospective studies would investigate if appropriate upkeep of suitable VDSL can decrease the risk of primary and recurrent melanoma diagnosis.

## Introduction

Cutaneous melanoma is epidermal neoplasia with the tendency to early invasion. The majority of melanoma involves the epidermis. The molecular diversity between various melanoma can significantly modify their outcome and their response to treatment. Despite the recent progress, the prevention of metastatic melanoma is a sphere still under study. The prognosis for patients with widespread metastasis remains poor^1–3^; early detection requires the research of new tools for the prevention of this neoplasia.

Long ordered to prevent and cured bone-related diseases, vitamin D has been considered in recent years as a potential tactic to forestall cancer and metabolic diseases.

Moreover, some studies demonstrate lower mortality in patients affected by cancer and cardiovascular disease in regions with more durable sun exposure, which is an important factor for epidermal creation of vitamin D^4,5^. CYP2R1, CYP27A1 and CYP27B1 hydroxyl vitamin D to produce 1,25(OH)2D3, or CYP11A1 to produce mono-di- and trihydroxy-D3 forms. Vitamin D3 activated has various functions, including a photo-protective role versus UVB-induced damage. These effects take place through an interaction with the vitamin D receptor (VDR) and the action on retinoic acid orphan receptors α and γ. On the contrary, mutation of the vitamin D-binding protein (VDP) and the VDR genes facilitate insurgence of melanoma^6,7^. Furthermore, other studies highlighted associations between low serum levels of 25-hydroxyvitamin D and increased risks of cancer and cardiovascular disease^8,9^.

The purpose of this study is to deepen the role of vitamin D in melanoma, that might provide new therapeutic and preventative methods.

## Patient and methods

The following research is an observational study. Population under examination is represented by patients who received a diagnosis of cutaneous melanoma in Varese from 2016 to 2019. Within this population, we have selected exclusively the patients for whom we were able to derive serum vitamin D levels at diagnosis of melanoma. The patients were operated at the U.O. of Dermatology and Plastic Surgery of the Circolo Hospital and refer to the Dermatology department for follow-up. Study subjects were selected retrospectively, starting from the nominal archive of melanoma follow-up. In case selection not restrictions on sex, age or ethnicity were applied. The clinical and personal details of the patients were obtained from the medical chart and reports on the Department Portal. The histopathological data of the melanoma were obtained from the reports of the OU of Anatomic Pathology of the Circolo Hospital. In the case of patients with multiple melanomas, this data was specifically reported and in the data analysis the neoplasm was associated with major malignancy (more advanced stage or Breslow’s thickness).

In case of indication for sentinel lymph node biopsy, we retrieved from the anatomopathological report the data about the positivity or negativity of the lymph node for metastasis.

In the case of emptying of the involved lymph node station, we reported the positivity or negativity of the lymph nodes removed. The clinical and demographic parameters analyzed for each patient are: gender and date of birth, family history of melanoma, date of diagnosis, age at diagnosis, years of survival from diagnosis, possible death, any previous or subsequent melanomas, location of the neoplasm, stage of malignancy (pTNM), possible sentinel lymph node biopsy and histological result, possible emptying of the lymph node station and histological outcome, any request for genetic testing, multiple melanomas.

The histopathological parameters of the tumour considered are: Breslow thickness, mitotic index (mitosis/mm^2^), Clark level, histotype.

For each patient, we measure out 25OH Vitamin D3 serum levels (ng/ml) by liquid chromatography tandem mass spectrometry at the diagnosis of melanoma. The dosage of vitamin D is requested at each clinical check during the follow-up for melanoma. However, if available, data required for other reasons have also been taken into consideration if temporally closer to diagnosis.

We requested the search for mutations of the BRAF, CDKN2A, CDK4 genes in case of familiarity for melanoma and in patients who have undergone adjuvant therapy.

The exclusion criteria from the study are:

- The time distance between the dosage of vitamin D and the diagnosis of melanoma greater than six months;
- Patients receiving vitamin D supplementation at the time of diagnosis.

Follow up of patients affected by melanoma was performed following AIOM guidelines 2018 and the experimental protocol was approved by the University of Insubria.

A control group, consisting of 125 patients with a negative history of melanoma, was then recruited for the dosage of vitamin D to the outpatient U.O.C. of the Clinical Chemical Analysis Laboratory of the Circolo Hospital and Foundation Macchi.

All patients recruited in our study signed an informed consent for the employment of personal data.

We used the 2009 7th of the AJCC Staging Manual for the staging of melanoma. The indication for sentinel lymph node biopsy was staging ≥ T1b, thus including thin melanoma with mitosis, according to the 7th edition.

Statistical analyses were performed using MedCalc (Version 19.0.6 of 2019). The significance level was set at 0.05. Any value obtained significantly lower than 0.05 (*p* < 0.05) was considered statistically significant.

## Results

The population consisted of 154 patients, 78 males (50.6%) and 76 females (49.4%). The average age at diagnosis of melanoma is 59.7 ± 15.5 years, slightly higher in men than women (62.6 and 54.8 years respectively). In this three-year period, 4 patients died (2.6%, 3 males, 1 female). Positive family history for melanoma was observed in 10/154 patients (6.5%). Localization and gender distributions are described in Table 1.

**Table 1:** Localizations and gender distribution.

Location	M–F		Male		Female	
	Number	%	Number	%	Number	%
Trunk	75	48.7	46	59.0	29	38.2
Lower Limbs	40	26.0	13	16.7	27	35.5
UpperLimbs	27	17.5	12	15.4	15	19.7
Head	12	7.8	7	9.0	5	6.6

The distribution of melanoma’s histotype is described in Table 2.

**Table 2:** Histotype's distribution.

Histotype	Number of patient	%
SuperficialDiffusion	121	78.6
Nodular	17	11.0
AcralLentiginous	3	1.9
Lentigo maligna	1	0.6
Others	12	7.8

Others histological parameters:

- Breslow thickness: the average thickness is 1.88 ± 2.56 mm, with a maximum value of 17 mm and a minimum value of 0.1 mm, attributable to melanomas in situ.
- Ulceration: 23/154 melanomas are ulcerated (14.9%), compared to 131 non-ulcerated melanomas (85.1%).
- Mitotic index: 84/151 patients present mitosis (55.6%), against 67 cases in which mitosis is not identified. The average of the population is 3.1 ± 6.0 mitosis/mm^2^.
- Clark level: considering 151 patients, we detected 3 melanomas of level I, 45 of level II, 50 of level III, 42 of level IV and 11 of level V.

Regarding the neoplastic staging, at the time of diagnosis, 104/152 melanomas (68%) were identified in stage 1, followed by neoplasms in stage 2 (23/152; 15.1%), stage 3 (22/152; 14.5%), stage 0 (2/152; 1.3%), and stage 4 (1/152; 0.7%).

Biopsy and histopathological analysis of the sentinel lymph node was performed in 71/152 patients (46.7%) and it was positive in 16 cases (22.5% of the operated).

Surgical dissection of the lymph node station was performed overall in 17 patients. Following the pathological analysis, the lymph nodes removed gave a positive result for metastases in 6 cases (35.3%).

The research for mutations in patients with melanoma was carried out in 31/154 cases (20.1%) for two main reasons:

- To identify a mutation of CDKN2A or CDK4 genes in 4 patients with family history positive for melanoma following genetic counselling;
- Perform a mutational analysis of BRAF to set up any adjuvant therapy. We researched this mutation in 27 patients and 12 cases were positive (44.4%).

We stratified our population on the vitamin D serum levels (VDSL). The results are described in Table 3.

**Table 3:** VDSL: vitamin D serum levels.

VDSL	Melanoma		Controls group	
	Number	%	Number	%
Several deficit (< 10 ng/ml)	20	13	7	5.6
**Deficit**				
(10–20 ng/ml)	56	36.4	17	13.6
Sufficiency (20–30 ng/ml)	56	36.4	40	32.0
Desirable (30–100 ng/ml)	21	13.6	61	48.8
**Toxicity**				
(> 100 ng/ml)	1	0.6	0	0.0

### Statistical analysis

The primary endpoint of the study was to evaluate whether low or high VDSL represent a risk factor for the development of melanoma. We compared the prevalence of vitamin D deficiency in the two populations, using the Fisher exact test.

At the time of diagnosis 76/154 patients (49.4%) had deficient vitamin D levels, compared to 24/125 subjects (19.2%) belonging to the control group. The prevalence of deficient vitamin D levels was significantly higher in the group of patients with melanoma (*p* < 0.000001).

The calculation of the Odds Ratio showed a moderate negative association between melanoma and VDSL > 20 ng/ml (OR = 0.24; 95% CI 0.14–0.42; *p* < 0.0001)**.** We repeated the calculation of the Odds ratio by comparing the number of patients with the deficit (< 20 ng/ml) and with desirable levels (> 30 ng/ml) in the two groups. A moderate negative association was demonstrated between melanoma and desirable levels of vitamin D (OR = 0.11; 95% CI 0.06–0.22; *p* < 0.0001).

**Figure 1:**
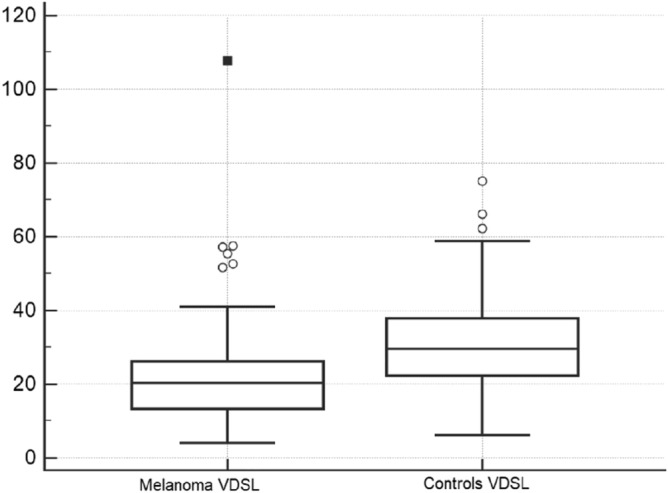
Comparison of VDSL (vitamin D serum levels) in melanoma (n. 154 subjects) and control group (n. 125 subjects) *p* < 0.0001.

We compared the medians of VDSL in the two groups using the Mann–Whitney Test: the median of the group of patients with melanoma was significantly lower than the control group (*p* < 0.0001, Fig. 1).

In the second part of the data analysis, we focused on the relationship between vitamin D and clinical-prognostic factors of neoplasia. We eliminated from our population 6 outliers identified by the Tuckey Method. The distribution was similar to a Gaussian curve (confirmed by the Shapiro–Wilk Test; *p* = 0.0568).

**Figure 2:**
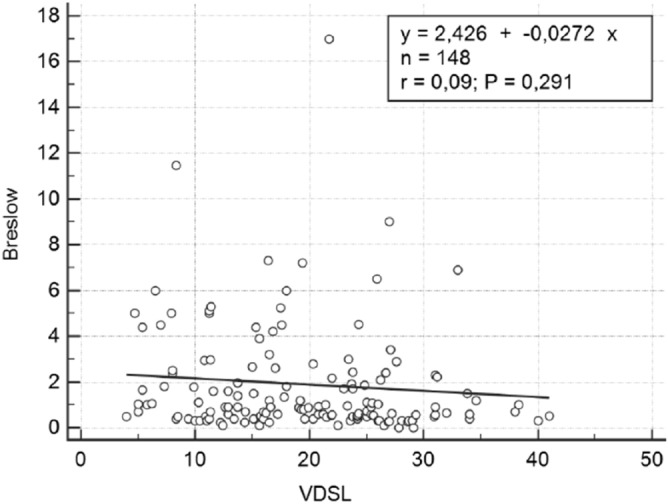
Linear regression between Breslow and VDSL (vitamin D serum levels). 148 subjects, *p* = 0.291.

To identify a relationship between Breslow thickness and vitamin D levels, we used the linear regression method. Breslow thickness was the dependent variable and VDSL was the independent variable. No significant correlation between the two elements was found (*p* = 0.2914, Fig. 2).

We divided the population according to the thickness of Breslow into five groups (thickness < 0.75 mm/0.75–1 mm/1–2 mm/2–3 mm / > 3 mm), and compared the averages of the VDSL by performing the analysis of variance with ANOVA test: VDSL in the groups do not differ significantly (*p* = 0.094).

**Figure 3:**
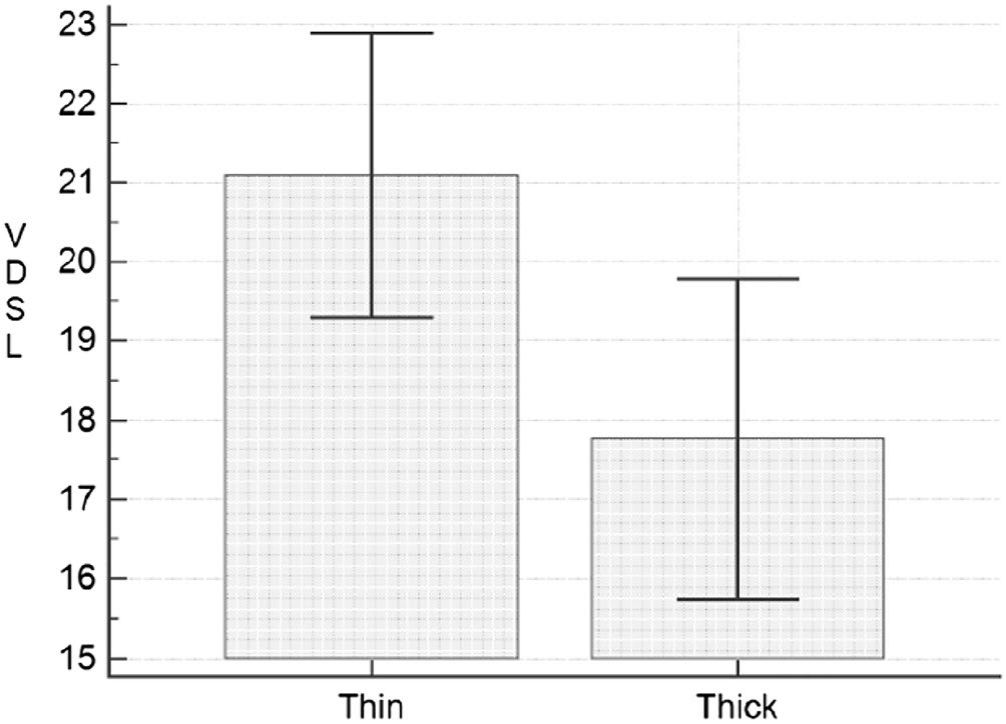
Comparison of VDSL (vitamin D serum levels) between patients with thin (< 1 mm, 83 subjects) and thick (> 1 mm, 65 subjects) tumors. *p* = 0.0154.

We compared the average VDSL between patients with thin tumour (thickness < 1 mm) and thick tumour (thickness > 1 mm) with the T-Test of Student. We found a statistically significant difference between the two groups (21.1 ± 8.2 ng/ml vs 17.8 ± 8.1 ng/ml; *p* = 0.0154; Fig. 3).

**Figure 4:**
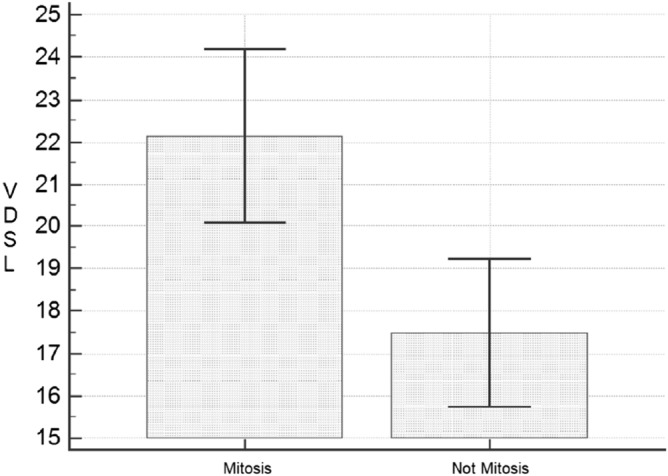
Comparison of VDSL (vitamin D serum levels) between patient with mitotic (80 subjects) and non mitotic melanoma (65 subjects). *p* = 0.0007.

The relationship between vitamin D and the presence of ulceration was investigated by Student's T-test: no significant difference was identified (19.9 ± 8.5 ng/ml vs 17.9 ± 7.3 ng/ml; *p* = 0.3204). We compared the average VDSL in patients with melanoma in which mitosis were not identified (22.1 ± 8.3 ng/ml), to those that had mitosis (17.5 ± 7.8 ng/ml) with Student's T-test. A statistically significant difference was observed (*p* = 0.0007, Fig. 4).

We divided the population based on VDSL (deficit, sufficiency, desirability), comparing the prevalence of mitotic activity in the three groups, by Chi-Square test: we found 49/75 (65.3%) melanomas with mitotic activity in patients with deficiency of VDSL, 26/54 (48.1%) in the group with sufficient VDSL and 8/21 (38.1%) in subjects with desirable VDSL.

In this case, the difference between the three groups was statistically significant (*p* = 0.0353).

We examined the relationship between the average of VDSL and the histotype, performing the ANOVA test: no difference was noted (*p* = 0.161).

We subsequently studied the relationship between the average of VDSL and levels of Clark with ANOVA test by dividing the population into five groups based on Clark's level. We did not found a statistically significant difference in VDSL (*p* = 0.371).

Looking for a relationship between VDSL and the site of onset of melanoma we stratified the population according to the site of the neoplasm (trunk, head, upper limbs, lower limbs) and we compared the VDSL average in the different groups with ANOVA test. We did not detect differences statistically significant (*p* = 0.630). To study the relationship between the average of VDSL and the melanoma staging, we performed the ANOVA test (stages 0 and 4 have not been considered). No significant difference was observed in the three groups (*p* = 0.130).

In order to study the relationship between VDSL and positivity or negativity sentinel node, we performed Student's T-test. In patients with negative sentinel node, the average VDSL was 19.2 ± 9.0 ng/ml, while in subjects with a sentinel node positivity the average was 18.4 ± 6.9 ng/ml (*p* = 0.7385).

Finally, to check for any difference in the average VDSL in the two genders, we performed Student's T-Test (20.4 ± 7.8 ng/ml vs 18.9 ± 8.8 ng/ml; *p* = 0.2711). We did not found a statistically significant difference.

## Discussion

In recent years, the link between VDSL and chronic diseases and neoplasms became an object of interest^10–12^. However, the relationship between VDSL and melanoma is not clarified^13^. The purpose of our study is to verify whether there is a relationship between VDSL at diagnosis and the risk of developing melanoma. In our population, the prevalence of vitamin D deficiency in patients with melanoma is significantly higher than the control group. At the time of diagnosis, compared to control group in which only 19.2% have VDSL lower than 20 ng/ml, 49.4% of the population with melanoma is in a state of vitamin D deficiency.

The calculation of the Odds Ratio also demonstrated a moderate negative association between sufficient and desirable values of vitamin D and neoplasm, allowing us to hypothesize that an optimal state of vitamin D may play a protective role towards melanoma. The observed data are aligned with the results of studies that identified low VDSL as a risk factor for melanoma development. The comparison of our results to the Cattaruzza and Nurnberg studies are summarized in Table 4 (Fisher's Exact Test)^10,12^.

**Table 4:** Comparison of vitamin D serum levels (VDSL) in Varese study (V), Cattaruzza (C) and Nurnberg (N).

VDSL	M						C						*p*		
	n			%			n			%					
	V	C	N	V	C	N	V	C	N	V	C	N	V	C	N
≤ 20 ng/ml	76	91	160	49.4	66.2	78.1	24	15	89	19.2	15.2	63.1	< 0.001	< 0.001	< 0.001
> 20 ng/ml	78	46	45	50.6	33.8	22.0	101	84	52	80.8	84.8	36.9			
≥ 30 ng/ml	22	10	–	14.3	7.3	–	61	37	–	48.8	37.4	-			

Study	Mediancases (ng/ml)	Median controls (ng/ml)	*p*												
Varese	20.3	29.5	< 0.0001												
Cattaruzza	18.0	27.8	< 0.001												
Nurnberg	16.9	18.8	0.44												

Finally, after excluding outliers in both populations, we have observed that even the average levels of vitamin D in patients with melanoma differ statistically significantly from the control group. The average serum vitamin D levels in the population with melanoma (19.6 ± 8.3 ng/ml) is significantly lower than that found within the healthy population (29.7 ± 11.0 ng/ml).

In this study, we analyzed the relationship between VDSL and Breslow by different tests, obtaining some significant results that indicate an association between low values of vitamin D and greater tumour thickness. The results found are aligned with the ones of Lim Newton-Bishop and Randerson-Moor studies^14^–^17^].

Considering the two classes of patients with Thin and Thick tumours, VDSL were significantly lower in subjects with Thick tumour (17.8 ± 8.1 ng/ml vs 21.1 ± 8.2 ng/ml). The same analysis was carried out in Lim's study. They obtained a similar result with lower vitamin D levels in patients with melanoma thicker than one millimetre.

Subsequently, after stratifying the population into five groups according to the Breslow, we compared the averages of the VDSL. Even if no significant difference was identified between the various groups, the highest VDSL was found in patients with lower Breslow thickness (< 0.75 mm). The same evaluation was performed, with statistical significance, in the Lim, Newton-Bishop and Randerson-Moor study^17^–^19^.

Even if we did not find statistical significance, the VDSL in ulcerated melanomas we lower the nonulcerated melanomas. The same scenario is outlined in Lim's study. However, in this case, the difference between the two groups was statistically significant.

Furthermore, we found a significant association between VDSL and the presence of mitosis. Mitotic index of 1/mm^2^ is considered, by the staging protocol of the American Joint Cancer Committees, an important threshold from the prognostic point of view. By stratifying the population on VDSL, we found that the prevalence of melanomas with mitosis in patients with vitamin D deficiency reached 65.3%, which is significantly higher than the group of subjects with sufficiency (48.1%) and with desirable levels (38.1%).

Moreover, we ascertained that VDSL was significantly higher in the group of patients with mitosis-free melanomas. The role of vitamin D within the processes of regulating cell growth could justify these results. In fact, Vitamin D inhibits proliferation and promotes apoptosis and cell differentiation. The same analysis was performed in the Lim study which identified a higher VDSL in non-mitotic melanomas (27.2 ng/ml vs 22.8 ng/ml, *p* = 0.036).

In our study, even if statistical analysis did not detect a significant difference among VDSL and different histotypes of melanoma, we observed that the lowest VDSL is related to the nodular melanoma. Although the histotype is no longer considered a prognostic factor, it is important to underline that nodular melanoma is characterized by a vertical growth and represents a negative prognostic factor. Indeed, it is associated with a high probability of development of loco-regional or remote metastasis.

We then considered the relationship between vitamin D levels and the Clark level of neoplasm. Through an analysis of the results, lower VDSL (19.1 ± 8.4 ng/ml) corresponds to the group of patients with level IV of Clark. Higher VDSL was found in patients with Clark level I (27.8 ± 1.3 ng/ml). However, we found no significant difference in the diverse groups.

Even in the Lim study, no significant association was identified.

We compared the average values of vitamin D in patients with malignancy in different stages. The comparison was implemented considering the patients with stage 1, 2 and 3 melanoma. VDSL is higher in the group of patients with stage 1 melanoma (20.4 ± 8.4 ng/ml), lower in the stage 2 population (18.1 ± 8.8 ng/ml) and decreases further in patients with stage 3 melanoma (16.8 ± 7.1 ng/ml). Albeit a statistically significant difference between the groups is not appreciable.

In the study conducted by Nurnberg et al., a statistically significant difference was highlighted among VDSL in stage 1 patients compared to stage 4 patients. On the other hand, in Lim's study that compared vitamin D levels in patients with melanoma in a non-metastatic and metastatic state, was not found a statistically significant difference in the two groups.

Besides, we looked for a correlation between VDSL and positivity sentinel node, if a biopsy had been performed. Even if there was not a statistically significant result, the group of patients with negative sentinel lymph node had VDSL higher than the population with sentinel lymph node positivity for melanoma metastasis (19.2 ± 9.0 ng/ml vs 18.4 ± 6.9 ng/ml).

Our study was conducted considering the patients belonging to the surgery follow-up melanoma of the Dermatology department; this resulted in one underestimation of the prevalence of stage 4 malignancies. Numerous patients, following the diagnosis of advanced melanoma, continued the therapeutic *iter* and follow-up in an oncological environment or in other specialized centers.

The prevalence of stage 0 malignancies may also be underestimated since, in line with AIOM 2019 guidelines^20^, the visit, for patients with melanoma in situ, is fixed one year after the intervention.

Being a retrospective study, it was not possible to collect other information that could influence the risk of melanoma and the state of vitamin D, such as BMI, skin type, seasonality of the dosage and sun exposure throughout life.

Why does vitamin D have protective effects? The reason has been extensively investigated.

Many studies emphasize the skill of this secosteroid to inhibit proliferation and stimulate the differentiation of melanoma cells that produce vitamin D receptors. Moreover, other studies suggest that vitamin D limits invasion and angiogenesis of tumour cells. Furthermore, VDR genetic mutations correlate with an increased risk to incur in melanoma and with a graver prognosis^21,22^.

Finally, the trend that emerges in this study recognizes lower VDSL in association with negative prognostic factors and advanced stage neoplasia.

**Figure 5:**
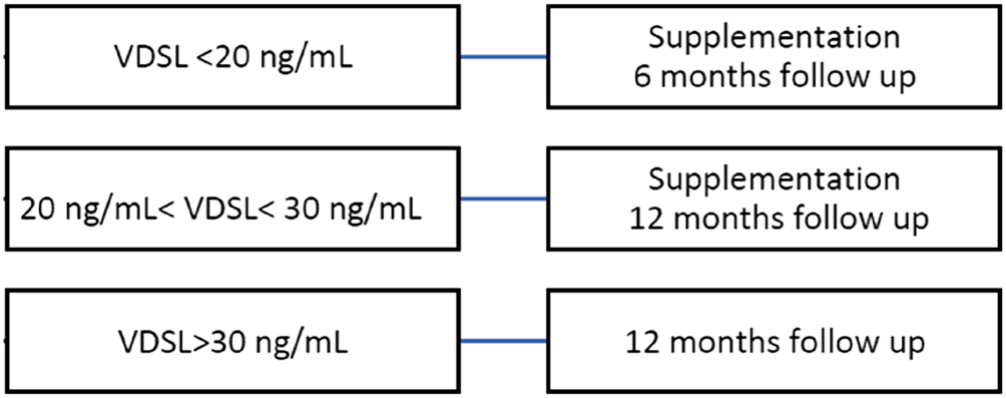
Vitamin D surveillance and melanoma.

Taking into consideration the AIOM 2019 guidelines, the results of our center and other groups mentioned in this study, we recommend the following flow chart to supervise the VDSL upkeep in patients with risk factors for melanoma (familiarity, recent melanoma, phototype I) (Fig. 5).

## Conclusion

From the comparison between the population with melanoma and the control group, we have identified significantly lower VDSL in the first group. This result agrees with other studies that recognize a deficiency status of vitamin D as a possible predisposing factor for the development of melanoma. The hypothesis is supported by the deficit of the known anti-proliferative and antiangiogenics effect attributable to calcitriol, which would have an antitumor action. Future perspective should investigate the possible correlation between novel pathways of vitamin D3 and 7-dehydrocholesterol metabolism initiated by CYP11A1 and the melanogenesis^23^.

However, it is important to underline that, being a cross-sectional study, we could consider the prevalence and the association of this vitamin with the neoplasm, without being able to verify the actual existence of one cause-effect relationship between the two elements. Optimal levels of vitamin D are a mirror of a healthy lifestyle (regular physical activity, diet adequate, BMI in the norm) and, therefore, could simply represent a surrogate marker for other protective factors against the development of malignancies. In the future, it may be interesting to carry out new case-control studies, designed on a larger population, aimed at analyzing risk factors for melanoma and their relationship with vitamin D. In this way it would be possible to define accurately the relationship between the vitamin and the risk of cancer. Another possible perspective could be to investigate whether, through adequate supplementation, the maintaining desirable vitamin D values may reduce the risk of relapses in patients with previous melanoma.

Take-home messages:

- Low dose vitD associates with thicker melanomas;
- Low dose vitD associates with a higher mitotic rate;
- VitD synthesis requires UV radiation, a known risk factor for melanoma. Dietary supplementation may be helpful.
